# Detroit, September, 1900

**Published:** 1900-07

**Authors:** 


### A. V. M. A.

### DETROIT, SEPTEMBER, 1900.

Prof. R. S. Huidekoper will visit Toronto, Canada, before reaching Detroit, and there fill the rôle of judge of several classes of horses at the great exhibition held there early in September.

Editor Bell, of the *Review*, will reach Detroit by a roundabout trip through Virginia, where he will spend several days roaming over his rural estate, and will be fully prepared to discuss crops as well as every practical veterinary subject.

Prof. A. Smith, of Toronto, Canada, so active in the work of the great fair held in Toronto in September, will forego his duties for a day and visit the convention at Detroit.

President Pearson has selected Dr. W. Horace Hoskins, of the JOURNAL staff, to respond to the Mayor's welcome at Detroit.

